# Perspectives on the Current Role of Radiation in the Management of Gastroesophageal Adenocarcinomas

**DOI:** 10.7759/cureus.109642

**Published:** 2026-05-25

**Authors:** Pallavi S Kapilavaih, Jacob Luddington, David A Saez, Neil Newman

**Affiliations:** 1 Radiation Oncology, UT Health San Antonio, San Antonio, USA

**Keywords:** chemotherapy, esophageal adenocarcinoma (eac), esophageal squamous cell carcinoma (scc), esophagectomy complications, neoadjuvant chemoradiotherapy

## Abstract

Over the past two decades, management approaches for esophageal and gastroesophageal junction (GEJ) cancers have undergone a notable transformation, driven by clinical trials aimed at optimizing the use of surgery, chemotherapy, and chemoradiotherapy (CRT) in treatment. Landmark studies have confirmed the essential role of neoadjuvant CRT (nCRT) and perioperative chemotherapy compared with surgery alone in improving survival rates and pathological complete response (pCR). Recently, active surveillance has emerged as a safe alternative to immediate surgery, and in select patients, adjuvant immunotherapies have demonstrated enhanced efficacy while reducing complications. This review will discuss the data that have helped shape the current treatment landscape for esophageal and GEJ cancers, as well as potential future directions.

## Introduction and background

Esophageal cancer remains a major cause of cancer-related mortality worldwide [1]. It is estimated that approximately 500,000 new cases of esophageal cancer are diagnosed each year, including 22,000 in the United States [1,2]. Histologically, esophageal cancers are classified into two main types: adenocarcinoma and squamous cell carcinoma (SCC). SCC, associated with tobacco and alcohol use, is more prevalent worldwide, whereas adenocarcinoma, associated with gastroesophageal reflux disease, obesity, and Barrett's esophagus, is more common in Western nations [2].

In the past, curative management of adenocarcinoma and SCC focused on esophagectomy. However, this strategy resulted in low five-year survival, high recurrence rates, and complications such as anastomotic leak and pulmonary issues, as well as a diminished quality of life [2,3], highlighting the need for improved management strategies. Treatment of esophageal cancer has undergone substantial evolution over the past several decades, reflecting a growing recognition that surgery alone is insufficient to achieve adequate local control and survival. A growing body of clinical research supports combining chemotherapy and chemoradiotherapy (CRT) with surgery [4].

Among the earliest of these efforts was the CROSS (Chemoradiotherapy for Oesophageal Cancer Followed by Surgery Study) trial, which established the benefits of neoadjuvant CRT (nCRT) for the treatment of esophageal and gastroesophageal junction (GEJ) adenocarcinomas and SCC. The study’s treatment regimen, consisting of radiation and doublet chemotherapy administered before surgery, demonstrated significant improvements in overall survival (OS), margin-negative (R0) resection rates, and pathological complete response (pCR) compared to surgery alone. The findings confirmed a clear benefit of CRT, leading to the adoption of the CROSS regimen as a new global standard for treating resectable esophageal and GEJ adenocarcinomas and SCC [5]. Similarly, the POET (PreOperative therapy in Esophagogastric adenocarcinoma) Stahl trial reaffirmed CRT’s effectiveness, this time compared with neoadjuvant doublet chemotherapy alone, showing superior results in OS, pCR, and local recurrence [6].

Subsequent advances have continued to shape treatment guidelines, with contemporary investigations supporting an active surveillance approach that aims to maximize survival while minimizing the complications of esophagectomy in select patients. In this review, we will contextualize the recent data, with an emphasis on adenocarcinoma, to examine the optimal treatment approaches in light of several new randomized trials.

## Review

Methodology

A literature search was conducted primarily using PubMed, Google Scholar, Scopus, and Web of Science databases. Search terms included “esophageal adenocarcinoma,” “chemoradiation,” “radiation therapy,” “neoadjuvant,” “definitive CRT,” and “clinical trial.” Prospective clinical trials were considered for inclusion based on their impact on standards of care, citation frequency, and relevance to the role of CRT in esophageal adenocarcinoma treatment. Selection involved an initial screening of titles and abstracts, followed by full-text assessment. Data extracted included trial design, patient population, treatment regimens, and primary outcomes, such as overall survival, pathologic response, and treatment-related toxicity.

Chemoradiotherapy (CRT) + surgery vs. surgery alone

The landmark 2012 CROSS trial included 366 patients (75% adenocarcinoma and 23% SCC) presenting with a T1N1 or T2-T3 N0-1 tumor. Patients were randomized to receive CRT consisting of carboplatin, paclitaxel, and concurrent radiation of 41.4 Gy in 23 fractions using an opposed-field technique, followed by esophagectomy, or to the control group treated by esophagectomy alone [7]. The treatment arm had a median OS of 49.4 months compared to 24.0 months in the control group (hazard ratio (HR): 0.657; 95% confidence interval (CI): 0.495 to 0.871; p = 0.003). Overall, pCR was 29% in the CRT group, with significantly higher rates for SCC at 49% than adenocarcinoma at 23% (p = 0.008) [8]. Furthermore, patients receiving CRT achieved an R0 resection rate of 92%, compared to 69% in the surgery-alone group (p < 0.001), and experienced significantly reduced locoregional recurrence rates at 14% versus 34% [8,9].

A follow-up published in 2015 further reinforced this benefit. Among patients with SCC, median OS reached 81.6 months in the CRT plus surgery group compared to 21.1 months for surgery alone (HR: 0.48; 95% CI: 0.28-0.83; log-rank p = 0.008). Although patients with adenocarcinoma experienced less dramatic gains, outcomes still favored CRT, with a median OS of 43.2 months versus 27.1 months in the control arm (HR: 0.73; 95% CI: 0.55-0.98; log-rank p = 0.038) [7]. This difference remains incompletely explained. However, esophageal SCC generally possesses enhanced sensitivity to chemoradiation compared to adenocarcinoma, likely as a result of multifactorial differences including distinct tumor microenvironments, molecular profiles, and locoregional patterns [10,11]. Improved outcomes for CRT with surgery over surgery alone were also reflected in the five-year OS, with 47% of patients alive compared to 34%, and in the 10-year OS, with 38% of patients alive compared to 25% [5,7]. 

Chemoradiotherapy (CRT) vs. neoadjuvant doublet chemotherapy

The 2009 POET Stahl trial aimed to determine whether CRT proved more effective than doublet chemotherapy as a neoadjuvant treatment, specifically for GEJ adenocarcinoma. The 119 evaluated patients in the study (uT3-4, NX, M0) were randomized to receive 2.5 cycles of cisplatin, leucovorin, and 5-fluorouracil (PLF) over 15 weeks or an initial 2.0 courses of PLF chemotherapy for 12 weeks followed by three weeks of chemoradiotherapy (30 Gy in 15 fractions with concurrent cisplatin and etoposide). All patients underwent esophagectomy after completion of preoperative treatment. Three-year survival was found to be 47.4% in the CRT arm compared to 27.7% in the chemotherapy-only arm (HR: 0.67; 95% CI: 0.41 to 1.07; log-rank p = .07). The trial closed early due to poor accrual and failed to reach statistical significance for the primary endpoint of three-year OS, but notable secondary measures favored the CRT arm. For example, 15.6% of CRT patients achieved a pCR versus only 2.0% in the chemotherapy-only arm (p=0.03), and 76.5% were free of local tumor progression at three years versus 59.0% (p=0.06) [6]. In conjunction with the CROSS trial, these findings established the benefits of trimodality therapy over bimodality therapy.

Chemoradiotherapy (CRT) vs. perioperative chemotherapy

Exploring a different strategy, the 2006 MAGIC trial demonstrated the survival benefits of adding perioperative chemotherapy to surgery for gastric and GEJ adenocarcinoma compared to surgery alone. As a result, epirubicin, cisplatin, and 5-fluorouracil (ECF), which the trial protocol intended to administer as three preoperative and three postoperative cycles, served as the chemotherapeutic standard for the treatment of gastric and GEJ adenocarcinoma [12]. This was until the 2017 FLOT4-AIO trial showed superior OS with the use of the 5-fluorouracil, leucovorin, oxaliplatin, and docetaxel (FLOT) regimen delivered as four preoperative and four postoperative cycles [13,14].

The 2014 Neo-AEGIS trial was a non-inferiority study that sought to reexamine the optimal treatment strategy for esophageal and GEJ tumors by investigating whether CRT or perioperative triplet chemotherapy yielded better survival outcomes. A total of 362 patients with cT2-3N0-3M0 esophageal or GEJ adenocarcinoma in 24 European sites were involved in the study. All patients underwent surgery after being randomly assigned to receive tri-modality therapy in the form of CROSS-established neoadjuvant CRT (carboplatin + paclitaxel + radiation) or ECF perioperative chemotherapy similar to MAGIC. Following the publication of the FLOT4-AIO trial, when FLOT became the standard of care, it was incorporated into the perioperative chemotherapy arm. CROSS-type neoadjuvant CRT revealed significant superiority over perioperative MAGIC/FLOT chemotherapy across several secondary endpoints, with a pCR of 12% compared to 4% (p = 0.012), nodal downstaging achieved in 60% of patients compared to 44% (p = 0.0035), an R0 resection rate of 96% compared to 82% (p = 0.0003), and a major pathological response observed in 39% of patients compared to 12% (p < 0.0001).

However, this did not translate into a survival advantage. Median OS was similar at 49.2 months and 48.0 months, and three-year survival rates were 57% and 55% in the tri-modality and perioperative chemotherapy groups, respectively (HR: 1.03; CI: 0.77-1.38; log-rank p = 0.82) [15]. It should be noted that although only 15% of patients in the chemotherapy arm received FLOT, the median OS in Neo-AEGIS was still higher than the 35 months reported among ECF patients in the FLOT4-AIO trial [13-15]. Similarly, the pCR rate in the CRT arm was lower than the 23% reported for adenocarcinoma patients in the original CROSS trial [8,15]. Furthermore, tri-modality patients experienced a lower incidence of Grade 3 neutropenia, 6% versus 27% among perioperative chemotherapy patients (p < 0.0001), and diarrhea, 0% versus 11% (p<0.0001). However, overall measures of quality of life, treatment-related toxicities, and postoperative complications did not significantly differ between the two arms.

The trial was only able to accrue 70% of the intended total participants, and after a futility analysis, the design was amended from testing a 10% superiority margin for the tri-modality therapy arm to assess non-inferiority of perioperative chemotherapy with a 5% margin for three-year OS. Additional enrollment would have been unlikely to alter the results [15]. Nonetheless, the study provides robust evidence that perioperative chemotherapy alone as a treatment strategy is comparable to tri-modality therapy in contemporary practice, and radiation is not always mandatory.

The 2017 TOPGEAR trial further explored the role of adding preoperative radiotherapy to chemotherapy in resectable gastric and GEJ cancers. It aimed to clarify whether incorporating radiation before surgery could improve outcomes beyond those achieved with chemotherapy alone. The study comprised 574 patients with primarily T3 gastric or GEJ adenocarcinoma who were randomized to the standard arm receiving perioperative ECF chemotherapy akin to MAGIC or the experimental arm receiving perioperative ECF chemotherapy and neoadjuvant CRT. Like Neo-AEGIS, most patients were treated with the ECF regimen and later with FLOT.

The TOPGEAR study concluded that the addition of neoadjuvant CRT did not confer a statistically significant survival advantage over chemotherapy alone, as the median overall survival rate was 46 months and 49 months, respectively (HR: 1.05; CI: 0.83-1.31). Postoperative complications and progression-free survival (PFS) also showed no statistically significant difference between the two arms, with a median PFS of 31 months for the CRT arm and 32 months for the perioperative chemotherapy arm (HR: 0.98; CI: 0.79 to 1.22). As seen in the Neo-AEGIS trial, measurements of local tumor control favored CRT. A higher percentage of patients in the CRT group had improved pCR, with 17% compared to 8%, and major pathologic response at 49.5% compared to 29.3% [16]. Despite these differences in local regional outcomes, no survival advantage was observed. 

Considering that patients were offered both FLOT and ECF in the Neo-AEGIS and TOPGEAR trials, it is difficult to interpret the individual efficacy of each regimen, particularly FLOT, given its established benefits, against CROSS CRT. This is what the 2024 ESOPEC trial wanted to clarify by directly comparing the efficacy of CROSS nCRT and perioperative FLOT chemotherapy. Radiotherapy was administered at a dose of 41.4 Gy with carboplatin and paclitaxel using a combination of three-dimensional conformal radiation (3D-CRT) and intensity-modulated radiation therapy (IMRT) techniques. Of note, the ESOPEC trial included higher-risk patients than the original CROSS study, with 6.6% having clinically staged T4 (cT4) and 79.7% being clinically node-positive (cN+), whereas no cT4 and only 64.5% cN+ patients were included in the CROSS trial. For the study’s 438 patients with esophageal adenocarcinoma, three-year OS was 50.7% with CROSS CRT and significantly improved with FLOT at 57.4% (HR: 0.70; CI: 0.53-0.92; p = 0.01).

The secondary endpoints generally favored FLOT. Patients receiving FLOT had a lower rate of isolated distant metastasis at 20.4% compared to 32.7% and demonstrated a higher pCR at 16.7% compared to 10.2% in CROSS patients. While isolated local progression, R0 resection, and grade 3 pathological tumor regression were inferior in FLOT than in CROSS at 7.7% versus 4.1%, 94.3% versus 95.0%, and 31.7% versus 19.0%, respectively. Also, only 50% of patients receiving CROSS experienced grade 3 or higher toxicity, while this was experienced by 58% of patients on the FLOT regimen [17]. These findings suggest that FLOT provides stronger systemic disease control, which translates to better survival rates. However, both CROSS and FLOT provided comparable local control, contrary to what was reported in the Neo-AEGIS and TOPGEAR trials [15-17].

In the ESOPEC trial, the CRT arm showed a median overall survival of 37 months, falling short of the 49.2 months seen in Neo-AEGIS and the 49.4 months originally reported in the original CROSS trial [7,15,17]. FLOT, on the other hand, exceeded expectations as median survival reached 66 months in ESOPEC, up from 50 months in the FLOT4-AIO trial and 47.2 months in the MATTERHORN control group [14,17,18]. These differences likely reflect factors such as trial population (CROSS included SCC patients and FLOT4-AIO included gastric cancer patients) as well as improvements in surgical care, like utilization of minimally invasive approaches to esophagectomy, and implementation of Enhanced Recovery After Surgery (ERAS) protocols [19,20].

Since adjuvant immunotherapy has increasingly become standard practice, a comparison between CROSS and FLOT-based perioperative chemotherapy under current treatment protocols is warranted. Additionally, subgroup analysis of FLOT-treated patients did not reveal notable differences in outcomes for those who were older than 60, had T2 disease, or Eastern Cooperative Oncology Group (ECOG) performance status greater than 0 (higher scores reflect greater limitations in daily function) compared to the primary analysis [17,21]. Because of the consistency of its benefits, FLOT is first-line therapy for resectable GEJ adenocarcinomas. For patients who are inoperable due to comorbidities or locally advanced disease, definitive CRT is the standard of care. Table 1 compares the findings of the key studies discussed thus far. 

**Table 1:** Findings of landmark clinical trials in the treatment of esophageal cancer OS: overall survival; pCR: pathological complete response; R0: margin-negative; CROSS: Chemoradiotherapy for Oesophageal Cancer Followed by Surgery Study; POET: PreOperative therapy in Esophagogastric adenocarcinoma; FLOT: 5-fluorouracil, leucovorin, oxaliplatin, and docetaxel; Neo-AEGIS: Neoadjuvant Trial in Adenocarcinoma of the Esophagus and Esophago-Gastric Junction International Study; TOPGEAR: Trial Of Preoperative therapy for Gastric and Esophagogastric junction AdenocaRcinoma; SCC: squamous cell carcinoma; GEJ: gastroesophageal junction; ECF: epirubicin, cisplatin, and 5-fluorouracil

Study name	Evaluated sample size	Histology	Arm A	Arm B	OS	pCR	R0
CROSS, 2012 [5,7,8]	366	75% adenocarcinoma, 23% SCC	Surgery alone	nCRT (carboplatin + paclitaxel + 41.4 Gy) → surgery	Significant in favor of nCRT	29% overall, 23% in adenocarcinoma, 49% in SCC	Significant in favor of nCRT
POET, 2009 [6]	119	GEJ adenocarcinoma	Neoadjuvant chemo (5-FU + cisplatin) → surgery	nCRT (5-FU + cisplatin + leuvoricin + 30 Gy) → surgery	No significant difference	Significant in favor of nCRT	No significant difference
MAGIC, 2006 [12]	503	Gastric/GEJ adenocarcinoma	Perioperative chemotherapy (ECF)	Surgery only	Significant in favor of ECF	Not reported	Significant in favor of ECF
FLOT4-AIO, 2017 [13,14]	716	Gastric/GEJ adenocarcinoma only	Perioperative chemotherapy (FLOT)	Perioperative ECF/ECX	Significant in favor of FLOT	Significant in favor of FLOT	Significant in favor of FLOT
Neo-AEGIS, 2014 [15]	377	Esophageal/GEJ adenocarcinoma	Perioperative chemotherapy (MAGIC/FLOT)	nCRT (CROSS)	No significant difference	Significant in favor of nCRT	Significant in favor of nCRT
TOPGEAR, 2017 [16]	574	Gastric or GEJ adenocarcinoma	Perioperative ECF chemotherapy	Perioperative chemotherapy + nCRT	No significant difference	No p-value reported 17% vs. 8% (favoring nCRT)	No p-value reported, 92.4% vs. 87.7% (favoring nCRT)
ESOPEC, 2024 [17]	438	Esophageal adenocarcinoma	Perioperative chemotherapy (FLOT)	nCRT (CROSS)	Significant in favor of FLOT	No p-value reported 16.7% vs. 10.1% (favoring FLOT)	No p-value reported 94.3% vs. 95.0% (favoring nCRT)

To further evaluate the ideal preoperative treatment for patients with GEJ adenocarcinoma, the RACE trial is currently being conducted to test whether adding CRT to a perioperative FLOT regimen is superior to perioperative FLOT on its own [22]. It is anticipated that this study will shed more light on the current role of CRT in the treatment of esophageal cancer. 

Immunotherapy as an extension to multimodal treatment

It should be highlighted that patients in the CROSS trial did not receive adjuvant immunotherapy if they achieved less than a pCR, as is now recommended per CheckMate 577 [7,23]. The study established nCRT followed by adjuvant nivolumab as the new standard of care for patients with residual disease. The CheckMate 577 study comprised 794 patients who had undergone CRT and esophagectomy with residual disease of at least ypT1 or ypN1 (yp indicates pathological stage after preoperative therapy) detected in the resected tissue. Both SCC and adenocarcinoma histology were included. Patients randomized to the treatment arm received nivolumab, and those in the control arm received a placebo.

The primary endpoint of median disease-free survival significantly favored patients receiving nivolumab at 22.4 months compared to the placebo recipients at 11.0 months (HR: 0.69; CI: 0.56-0.86; p < 0.001). The benefit was seen across nearly every subgroup regardless of patient age, histological type, ECOG score, pathological lymph node status, and expression of the biomarker PD-L1 (programmed cell death ligand). Grade 3 or 4 adverse events occurred in 13% of nivolumab and 6% of placebo recipients. The results of the Checkmate 577 trial demonstrate that immunotherapy doubles the time until recurrence in patients with residual disease and is generally safe [23].

PET response-adapted neoadjuvant therapy

Seeking to address concerns regarding high rates of distant failure after standard CRT, the CALGB 80803 trial examined whether positron emission tomography (PET) response could serve as a biomarker to tailor induction CRT and enhance outcomes in esophageal adenocarcinoma. The study comprised 225 patients who underwent PET evaluation with cT1N1-3M0 to T2-4 NanyM0 who were histologically confirmed to have adenocarcinoma of the esophagus or EGJ (Siewert types I and II). Of note, 83.0% had tumors of size T3 or T4, and 71.4% had N+ nodal involvement. All patients were randomized 1:1 to receive either modified oxaliplatin, leucovorin, and 5-fluorouracil (FOLFOX) or carboplatin-paclitaxel (CP). Historical data from studies that involved administration of CRT without response-based switching served as the control group. A PET scan was performed after initiating chemotherapy to classify patients as “PET responders” or “PET non-responders” based on tumor metabolic activity. PET responders continued their chemotherapy treatment, while PET non-responders were switched to the other regimen (FOLFOX to CP or CP to FOLFOX). Both groups then received 50.4 Gy of CRT before surgery. The primary endpoint for CALGB 80803 was pCR among PET non-responders after switching [24].

The median OS for PET responders was 48.8 months and 27.4 months in non-responders (p = 0.107). Of note, 64.9% of patients in the FOLFOX arm and only 56.1% in the CP arm were classified as PET responders, but this difference was not significant. PET responders who were continued on FOLFOX had the most favorable outcomes in survival, with a median OS that was not reached and a five-year OS of 53.0%. PET responders on CP had a lower median OS of 38.7 months and a five-year OS of 43.9%. Among PET non-responders, patients switched from FOLFOX to CP had a median OS of 28.7 months, while those switched from CP to FOLFOX had a median OS of 26.6 months. Among PET responders, the pCR rate was once again highest in the FOLFOX group at 40.3%, compared to 14.1% in the CP group (p=0.001). In PET non-responders, however, pCR was 20.0% (95% CI: 10.0-33.7) in those switched from CP to FOLFOX, and 18.0% (95% CI: 7.5-33.5) in those switched from FOLFOX to CP, which is substantially higher than the pCR of approximately 5% reported in historical non-switching cohorts [24]. Thus, suggesting that a change in therapy for PET non-responders, a group facing an otherwise unfavorable prognosis, improves their outcomes, akin to those in the PET responder group.

Surgical morbidity

The trials discussed thus far have focused on optimizing the treatment regimens used in conjunction with surgery. The impressive pCR rates seen with intensified neoadjuvant therapy have prompted greater consideration of nonoperative strategies for esophageal and GEJ cancer management. In rectal cancer, where high pCR rates can be achieved with CRT, an active surveillance approach for patients with complete clinical response has become an accepted alternative to immediate surgery, helping to avoid surgical risks and prolong quality of life [25-27]. Given these successes, active surveillance is now being explored in esophageal cancer as well, to determine whether surgery can be safely deferred in patients who achieve a complete clinical response. 

Worrell et al. analyzed 16,392 patients who had undergone an esophagectomy from the National Surgical Quality Improvement Program. The findings challenge the assumption that benign esophageal disease entails lower surgical risk. Among the study’s key findings was a major morbidity rate of 34.7% for benign esophagectomies, which was significantly greater than the 30.7% in malignant esophagectomies (p = 0.001), despite similar in-hospital mortality of 2.3% and 2.5% (p = 0.62) [28]. Complementing this, the 2023 Bonanno et al. study emphasizes the importance of patient-reported outcomes in assessing esophagectomy recovery.

Through survey data, it was found that patients experienced marked reductions in physical function and increases in pain interference and dyspnea within the first 50 days postoperatively. Although the difference from baseline was still statistically significant, a gradual recovery was observed at around 100 days. It is also notable that no major difference was found in physical function, pain interference, or dyspnea by six months among patients undergoing minimally invasive/robotic, transhiatal, and transthoracic esophagectomies [29]. Collectively, these studies underscore that while surgical resection remains a cornerstone of esophageal cancer management, tailoring care to patient needs is critical to improving quality of life.

Chemoradiotherapy (CRT) + active surveillance vs. CRT + surgery

Early studies exploring the need for surgery found similar survival rates between patients receiving CRT alone and CRT followed by surgery, providing evidence that non-surgical approaches might be feasible for select patients. Active surveillance for esophageal cancers is now the focus of ongoing studies. Among the studies providing initial support for surveillance was the 2005 Stahl JCO trial, which comprised 172 patients with SCC of T3-T4N0-1M0. Both arms received induction chemotherapy (5-fluorouracil + leucovorin + etoposide + cisplatin) followed by 40 Gy CRT. Patients were then randomized to receive either surgery or continue with a higher-dose CRT regimen of 65 Gy. Three-year survival rates for the surgery arm at 31.3% and 24.4% for the definitive CRT arm, and the median OS at 16.4 months and 14.9 months were equivalent. Improved local control was demonstrated by surgery, with a two-year PFS of 64.3% compared to 40.7% for definitive CRT (HR: 2.1; 95% CI: 1.3-3.5; p = 0.003). Although patients undergoing surgery experienced greater treatment-related mortality at 12.8% compared to 3.5% (p = 0.03) [30]. 

The 2007 Bedene JCO trial also compared CRT with and without surgery. The patients included had been diagnosed with T3N0-1M0 esophageal SCC and received induction CRT in either a conventional or split-course regimen. Patients were then randomized to receive either surgery or continue a more intense CRT regimen. The two-year OS was not significantly different between the two groups, with 34% in the surgery arm and 40% in the definitive CRT arm (p = 0.44) [31]. Akin to the 2005 Stahl JCO trial, local control measures were superior among surgery patients, with a two-year PFS of 64.3% compared to 40.7% in definitive CRT patients (p = 0.003) [30,31]. This was despite a similar frequency of metastases in both groups. It is also worth noting that mortality rates at three months were greater among surgery patients, at 9.3% versus 0.8% (p = 0.002), as well as at six months, at 16% versus 6% (p = 0.015) [31]. This suggests that for locally advanced esophageal SCC, when a response to chemoradiation is achieved, subsequent surgery improves local control but provides no additional benefit in OS.

The 2025 SANO trial is an ongoing study aimed at directly comparing the effectiveness of active surveillance to standard esophagectomy for patients that had achieved a complete clinical response after CRT. This phase 3 trial, taking place in 12 Dutch hospitals, includes 309 patients. A total of 198 patients were assigned to active surveillance, which involved surgery only when locoregional tumor growth was detected, and 111 underwent surgery. Unlike the Stahl JCO and Bedene JCO trials, SANO included both adenocarcinoma and SCC. Among the preliminary data reported was that the two-year OS rate for the active surveillance and surgery arms was not significantly different, with 74% and 71% of patients, respectively (HR: 1.14; 95% CI: 0.74-1.78) [32]. Further findings from the SANO trial may help establish the modern framework for evaluating active surveillance.

For patients with a complete clinical response, the best management may in fact be CRT followed by active surveillance, rather than immediate surgery, upending a decades-long mainstay of treatment.

## Conclusions

Over the past 20 years, several multimodal strategies have been explored in an effort to improve outcomes for esophageal and GEJ cancers. The CROSS and POET trials were among the first to establish the role of CRT in treatment alongside esophagectomy. While studies like Neo-AEGIS and TOPGEAR have compared perioperative chemotherapy regimens with trimodality therapy, showing differences in pCR and major pathological response, they have shown little separation in OS. More recent studies, such as CALGB 80803, have introduced response-adapted approaches using PET imaging to tailor chemotherapy to each patient. The high-yield pCR rates achieved from CRT have prompted a reexamination of the role of surgery in treatment for some patients. A current focus of the field is whether surgery can be avoided in patients who demonstrate a suitable clinical response to CRT. While the viability of this active surveillance method is still under investigation, it represents a shift in thinking from uniformly aggressive treatment to a more individualized balance between tumor control and quality of life.

## References

[1] Joseph A, Raja S, Kamath S (2022). Esophageal adenocarcinoma: a dire need for early detection and treatment. Cleve Clin J Med.

[2] Raymond DP, Seder CW, Wright CD (2016). Predictors of major morbidity or mortality after resection for esophageal cancer: a Society of Thoracic Surgeons General Thoracic Surgery Database Risk Adjustment Model. Ann Thorac Surg.

[3] Siegel RL, Kratzer TB, Giaquinto AN, Sung H, Jemal A (2025). Cancer statistics, 2025. CA Cancer J Clin.

[4] Shah RD, Cassano AD, Neifeld JP (2014). Neoadjuvant therapy for esophageal cancer. World J Gastrointest Oncol.

[5] Eyck BM, van Lanschot JJ, Hulshof MC (2021). Ten-year outcome of neoadjuvant chemoradiotherapy plus surgery for esophageal cancer: the randomized controlled CROSS trial. J Clin Oncol.

[6] Stahl M, Walz MK, Stuschke M (2009). Phase III comparison of preoperative chemotherapy compared with chemoradiotherapy in patients with locally advanced adenocarcinoma of the esophagogastric junction. J Clin Oncol.

[7] Shapiro J, van Lanschot JJ, Hulshof MC (2015). Neoadjuvant chemoradiotherapy plus surgery versus surgery alone for oesophageal or junctional cancer (CROSS): long-term results of a randomised controlled trial. Lancet Oncol.

[8] van Hagen P, Hulshof MC, van Lanschot JJ (2012). Preoperative chemoradiotherapy for esophageal or junctional cancer. N Engl J Med.

[9] Oppedijk V, van der Gaast A, van Lanschot JJ (2014). Patterns of recurrence after surgery alone versus preoperative chemoradiotherapy and surgery in the CROSS trials. J Clin Oncol.

[10] Gaber CE, Sarker J, Abdelaziz AI, Okpara E, Lee TA, Klempner SJ, Nipp RD (2024). Pathologic complete response in patients with esophageal cancer receiving neoadjuvant chemotherapy or chemoradiation: a systematic review and meta-analysis. Cancer Med.

[11] Dai YH, Chen PH, Huang YJ (2025). Radiation dose escalation in locally advanced oesophageal cancer: a systematic review and hierarchical Bayesian meta-analysis. EClinicalMedicine.

[12] Cunningham D, Allum WH, Stenning SP (2006). Perioperative chemotherapy versus surgery alone for resectable gastroesophageal cancer. N Engl J Med.

[13] Al-Batran SE, Homann N, Pauligk C (2019). Perioperative chemotherapy with fluorouracil plus leucovorin, oxaliplatin, and docetaxel versus fluorouracil or capecitabine plus cisplatin and epirubicin for locally advanced, resectable gastric or gastro-oesophageal junction adenocarcinoma (FLOT4): a randomised, phase 2/3 trial. Lancet.

[14] Al-Batran SE, Hofheinz RD, Pauligk C (2016). Histopathological regression after neoadjuvant docetaxel, oxaliplatin, fluorouracil, and leucovorin versus epirubicin, cisplatin, and fluorouracil or capecitabine in patients with resectable gastric or gastro-oesophageal junction adenocarcinoma (FLOT4-AIO): results from the phase 2 part of a multicentre, open-label, randomised phase 2/3 trial. Lancet Oncol.

[15] Reynolds JV, Preston SR, O'Neill B (2023). Trimodality therapy versus perioperative chemotherapy in the management of locally advanced adenocarcinoma of the oesophagus and oesophagogastric junction (Neo-AEGIS): an open-label, randomised, phase 3 trial. Lancet Gastroenterol Hepatol.

[16] Leong T, Smithers BM, Haustermans K (2017). TOPGEAR: a randomized, phase III trial of perioperative ECF chemotherapy with or without preoperative chemoradiation for resectable gastric cancer: interim results from an international, intergroup trial of the AGITG, TROG, EORTC and CCTG. Ann Surg Oncol.

[17] Hoeppner J, Brunner T, Schmoor C (2025). Perioperative chemotherapy or preoperative chemoradiotherapy in esophageal cancer. N Engl J Med.

[18] Janjigian YY, Al-Batran SE, Wainberg ZA (2025). Event-free survival (EFS) in MATTERHORN: a randomized, phase 3 study of durvalumab plus 5-fluorouracil, leucovorin, oxaliplatin and docetaxel chemotherapy (FLOT) in resectable gastric/gastroesophageal junction cancer (GC/GEJC). J Clin Oncol.

[19] Mann C, Berlth F, Hadzijusufovic E, Lang H, Grimminger PP (2020). Minimally invasive esophagectomy: clinical evidence and surgical techniques. Langenbecks Arch Surg.

[20] Low DE, Allum W, De Manzoni G (2019). Guidelines for perioperative care in esophagectomy: enhanced recovery after surgery (ERAS(®)) society recommendations. World J Surg.

[21] Azam F, Latif MF, Farooq A, Tirmazy SH, AlShahrani S, Bashir S, Bukhari N (2019). Performance status assessment by using ECOG (Eastern Cooperative Oncology Group) score for cancer patients by oncology healthcare professionals. Case Rep Oncol.

[22] Lorenzen S, Biederstädt A, Ronellenfitsch U (2020). RACE-trial: neoadjuvant radiochemotherapy versus chemotherapy for patients with locally advanced, potentially resectable adenocarcinoma of the gastroesophageal junction - a randomized phase III joint study of the AIO, ARO and DGAV. BMC Cancer.

[23] Kelly RJ, Ajani JA, Kuzdzal J (2021). Adjuvant nivolumab in resected esophageal or gastroesophageal junction cancer. N Engl J Med.

[24] Goodman KA, Ou FS, Hall NC (2021). Randomized phase II study of PET response-adapted combined modality therapy for esophageal cancer: mature results of the CALGB 80803 (Alliance) trial. J Clin Oncol.

[25] Garcia-Aguilar J, Patil S, Gollub MJ (2022). Organ preservation in patients with rectal adenocarcinoma treated with total neoadjuvant therapy. J Clin Oncol.

[26] Lai SH, Widmar M, Monson JR, Fleming FJ, Morris AM, Vogel JD (2025). Rectal cancer watch-and-wait management: experience of 545 patients from the US Rectal Cancer Research Group. Dis Colon Rectum.

[27] Valk MJM van der, Hilling DE, Bastiaannet E (2018). Long-term outcomes of clinical complete responders after neoadjuvant treatment for rectal cancer in the International Watch & Wait Database (IWWD): an international multicentre registry study. Lancet.

[28] Worrell SG, Gupta S, Alvarado CE, Sarode A, Luo X, Towe CW, Linden PA (2023). Morbidity after esophagectomy is higher for benign than malignant disease. Ann Thorac Surg.

[29] Bonanno A, Dixon M, Binongo J (2023). Recovery of patient-reported quality of life after esophagectomy. Ann Thorac Surg.

[30] Stahl M, Stuschke M, Lehmann N (2005). Chemoradiation with and without surgery in patients with locally advanced squamous cell carcinoma of the esophagus. J Clin Oncol.

[31] Bedenne L, Michel P, Bouché O (2007). Chemoradiation followed by surgery compared with chemoradiation alone in squamous cancer of the esophagus: FFCD 9102. J Clin Oncol.

[32] van der Wilk BJ, Eyck BM, Wijnhoven BP (2025). Neoadjuvant chemoradiotherapy followed by active surveillance versus standard surgery for oesophageal cancer (SANO trial): a multicentre, stepped-wedge, cluster-randomised, non-inferiority, phase 3 trial. Lancet Oncol.

